# A scoping review and thematic analysis of the landscape of spiritual health and spirituality in Canada

**DOI:** 10.1371/journal.pone.0340854

**Published:** 2026-02-20

**Authors:** Helana Marie Boutros, Merna Mina, Simon Lasair, Valerie Michaelson, Marilyn Baetz, Kristina Kokorelias, Maurita T. Harris, Nelly Van Doorn-Harder

**Affiliations:** 1 Department of Health, Aging & Society, McMaster University, Hamilton, Canada; 2 Faculty of Liberal Arts, Wilfrid Laurier University, Waterloo, Canada; 3 Department of Health Sciences, Wilfrid Laurier University, Waterloo, Canada; 4 Canadian Association for Spiritual Care/Association canadienne de soins spirituels (CASC/ACSS), Saskatchewan, Canada; 5 Robert Steane Holistic Research Chair, St. Paul’s Hospital Saskatoon, Saskatoon, Saskatchewan, Canada; 6 Faculty of Applied Health Sciences, Brock University, St. Catherines, Ontario, Canada; 7 Vice-Dean of Faculty Engagement in the College of Medicine, University of Saskatchewan, Saskatoon, Saskatchewan, Canada; 8 Department of Psychiatry, University of Saskatchewan, Saskatoon, Saskatchewan, Canada; 9 Royal University Hospital, Saskatoon, Saskatchewan, Canada; 10 Department of Occupational Science & Occupational Therapy, Temerty Faculty of Medicine, University of Toronto, Toronto, Canada; 11 Toronto Rehabilitation Institute, University Health Network, Toronto, Ontario, Canada; 12 Section of Geriatrics, Department of Medicine, Sinai Health Systems and University Health Network, Toronto, Canada; 13 Rehabilitation Sciences Institute, Temerty Faculty of Medicine, University of Toronto, Toronto, Canada; 14 Department for the Study of Religions, Wake Forest University, Winston-Salem, United States of America; University of Alabama at Birmingham, UNITED STATES OF AMERICA

## Abstract

Literature that focuses explicitly on *spiritual health as a dimension of health* remains comparatively underdeveloped in many national contexts, including Canada. Recent reviews published in 2007 and 2022 identify several persistent gaps in this area, particularly in how spiritual health is theorized, operationalized, and situated within broader frameworks of health. While the literature on *spirituality and health* is more substantial and has largely approached spirituality as *a determinant or influencing factor* shaping health outcomes, it nonetheless reveals conceptual and empirical limitations that call for further refinement. Taken together, these bodies of work point to the need for deeper and more sustained scholarly attention and greater conceptual clarity, particularly within Canada’s distinct spiritual and sociocultural landscape. For instance, Indigenous spiritualities in Canada, often rooted in Traditional Knowledge systems, intersect with broader discussions of spirituality and health. Furthermore, given Canada’s multicultural context, the convergence of religion with spirituality is also worth exploring. Covering over 30 years of research, this scoping review is arguably the first project of its kind to understand the landscape of both spiritual health and spirituality (in relation to health) in Canada. From an initial 4,977 articles collated, 187 articles met the inclusion criteria and were clustered around fifty primary health-related contexts. Collectively, our findings highlight where future research on this topic may be directed. This review also shares key thematic trends, namely the nuanced convergence of spirituality with religion concerning health; the other dimensions of health that spirituality is connected to or discussed with; the presence/role of interfaith dialogue; and the cultural considerations concerning spirituality and health. Overall, by moving beyond the descriptive content of our articles and mapping out the discursive content of the articles as well, this review not only fills critical gaps in Canada’s landscape of spiritual health, but also critically shows that Canada’s spiritual milieu is “thick” and “textured.” It is this nuance and complexity that we aim to elucidate in this scoping review.

## Background

Literature specifically addressing spiritual health remains limited across many countries, including Canada. The most recent reviews (2007 and 2022) identified several gaps in this field, including the absence of a consensus definition of spiritual health, limited integration of spiritual health within nursing education, insufficient attention to patient perspectives, and considerable variability in how spiritual practices are conceptualized and understood. [e,f in S2 File] These reviews also point to the need for more holistic and integrative approaches to spiritual health. By contrast, the broader literature on *spirituality and health* is comparatively more developed and has continued to expand within the Canadian context. However, this body of scholarship largely approaches spirituality in relation to health outcomes rather than engaging spiritual health as a dimension of health in its own right.

Taken together, these bodies of work point to the need for deeper and more sustained scholarly attention and greater conceptual clarity, particularly within Canada’s distinct spiritual and sociocultural landscape. Firstly, spirituality is central to Indigenous health, where First Nations, Métis, and Inuit peoples include spirituality as a critical dimension of their holistic health through their Traditional Knowledge systems. Secondly, Canada’s multiculturalism has rippled into what scholars like Parker (2018) call “multiple modernities,” which shapes not only one’s spiritual and cultural identities but also one’s spirituality in relation to health. [q in S2 File] This idea of “multiple modernities” rests on the following premise, namely that the process of secularization does not follow a unilateral trajectory across cultures and is influenced by religion. Furthermore, the secularization hypothesis itself is contested, with many theorists suggesting that we now live in a post-secular environment wherein religion is becoming more visible in the public sphere of post-secular Western societies, albeit in quite different and transformed forms than previously. [h, i in S2 File]

Overall, no reviews have synthesized knowledge specifically related to spiritual health and spirituality (as it pertains to health) in Canada to identify trends, themes, and gaps in the literature. As a result, this scoping review is arguably the first project of its kind. Covering over 30 years of research, this scoping review aims to understand the landscape of both spiritual health and spirituality (in relation to health) as they currently stand in Canada. Relatedly, this review also aims to understand the convergence of religion with spirituality and the extent of this convergence in the context of Canada, while addressing the methodological challenge of distinguishing between these often-overlapping concepts. Existing reviews primarily focus on “descriptive content,” which often overlook the underlying semantics through which spiritual health and spirituality (as it pertains to health) are understood and conceptualized. This tendency leads to an oversimplification of these concepts and diminishes the nuance of spiritual health and spirituality (as it pertains to health). To address this gap, the present review not only identifies this limitation, but also offers an ambitious synthesis of how spiritual health and spirituality (as it pertains to health) are shaped, articulated, and debated within contemporary Canadian literature. To our knowledge, this review is the first to go beyond sharing empirical findings and theoretical insights by critically examining the language and discourse behind these concepts as well. Finally, it is important to note that while this review does not focus explicitly on Indigenous health, Indigenous viewpoints, where relevant, have been prioritized and the dynamic tension between acknowledging Indigenous spiritualities and maintaining the scope of the review is navigated with care and sensitivity.

## Methods

Since the protocol for this scoping review has already been published, methods will only be *briefly* discussed. [b in S2 File] PRISMA-Scr guidelines were adopted and used to help standardize reporting (see table in S1 Table). [p,y in S2 File] Select electronic databases were searched in May 2024. Those included ProQuest Databases (including MEDLINE and APA PsycINFO), EBSCO Host Databases, Scopus, Embase, Web of Science, Anthropology Plus, and PubMed (see table in S2 Table). Our search strategy (see table in S2 Table) focused on three search strings, namely search terms related to “spirituality and spiritual health,” “health and well-being,” and the “Canadian context.” Religion was intentionally not included in the advanced search strategy to prioritize Canada’s current literature on spiritual health/spirituality while allowing the literature to *organically reveal whether and how* religion emerges as a related concept, without predetermining its role.

No restrictions were initially applied, except for limiting the search to English, peer-reviewed and Canadian-situated sources and, where available, using title or abstract filters offered by the electronic databases. Our inclusion criteria (see protocol) were carefully applied during the title/abstract screening and full-text screening phases to ensure relevance. HMB and MM initially screened titles and abstracts for eligibility, followed by a full-text review for articles already meeting preliminary criteria. A third reviewer, MTH, resolved any discordance between reviewers. Finally, to ensure the breadth and depth of our research scope, additional sources were identified by hand-searching articles via Google and sifting through the included articles’ reference lists. Guided by the ethos of this work, HMB and MM carefully reviewed hand-searched articles and reference lists, and engaged in ongoing discussions while applying judiciousness and discernment to determine what to include.

Each article, where appropriate, was extracted for methodology, data collection methods, study population, study context, and initial thematic characteristics. Using a reflexive thematic analysis approach, we undertook a preliminary reading of included articles and developed an initial thematic framework based on initial thematic characteristics that we opted to extract in our data extraction table. [b, c in S2 File] As data extraction and coding progressed, themes were iteratively discussed between HMB and MM during team project meetings.

While Arksey and O’Malley’s approach to scoping reviews offers a robust and widely adopted methodological framework, it does not explicitly account for the nuanced subjectivities that emerge within team-based review processes. Although adherence to the framework supports transparency and methodological rigor, decisions regarding inclusion and exclusion are often shaped through collaborative discussion and shared interpretation among team members. In the spirit of a team-based approach to scoping reviews, we engaged scholars throughout multiple stages of the scoping review. Notably, following data extraction, we consulted scholars from diverse disciplines, both authors of included studies and colleagues within our academic networks, to solicit feedback and identify any relevant works that may not have been captured in the initial search strategy. HMB, the project lead and conceptualizer of this work, met with each consultant individually to have real-time conversations and develop rapport before they agreed to participate. Once the manuscript was completed, an email thread was sent to all consultants requesting their review. To manage potential disagreements and avoid shoehorning specific viewpoints, we encouraged an open exchange of ideas in which each consultant could bring their unique disciplinary perspective without feeling constrained. This form of communication allowed consultants to engage with each other’s feedback and respond to one another’s comments. We hope that this consultative approach to scoping reviews may serve as a model for future knowledge dissemination.

Finally, this scoping review relied solely on secondary data and did not involve primary research with human subjects, so it did not require institutional ethical approval.

## Results

### Overview

**Fig 1:**
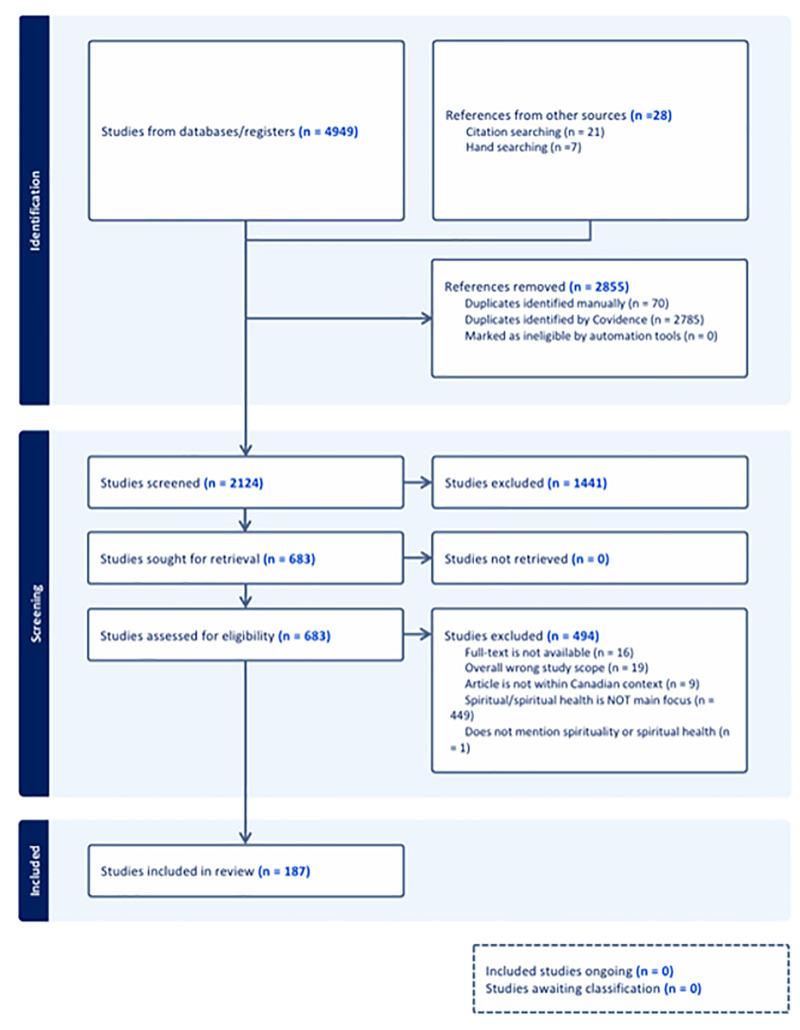
PRISMA flow diagram of the scoping review of spiritual health/spirituality in Canada.

Searches yielded a total of 4,977 citations (4,949 from databases/registers and 28 from other sources), of which 187 were included in this review (Fig 1). These articles were published between 1993 and 2024 and discussed spirituality as both a determinant and a dimension of health (see linked dataset). These citations were clustered around fifty primary contexts in which spiritual health/spirituality in relation to health was mentioned (see figure in S3 Fig). These primary contexts were clustered collaboratively and approved by the research team. Among these contexts, the largest number of studies focusing on spiritual health/spirituality in relation to health in Canada focused on Youth and Adolescent Health (n = 20); Psychiatry and Psychiatric Disorders (n = 18); Occupational Therapy (n = 14); Indigenous Communities (n = 11); Patient Care (n = 9); Spiritual Care Providers/Spiritual Health Practitioners/Chaplains (n = 9); Palliative and End of Life Care (n = 8); African and/or African-Caribbean Communities (n = 7). Remaining citations were distributed across 42 additional contexts.

Meanwhile, we captured key aspects of each article in addition to the primary context, which was organized as a separate column in our table (see “Contextual Specificities Alongside Primary Context” in linked dataset). For example, if an article focused on “Métis health,” it was recorded as a contextual specificity alongside the primary context of “Indigenous Communities.” Not only is this way of data extraction analogous to keyword designations in journal articles, but by avoiding pan-explaining or pan-reporting, it also provides a clear subject-based breakdown that improves informational accessibility for researchers and readers alike.

Of the 187 included citations, only 20 studies specifically used terminology like *spiritual health, illness, or distress.* We organized the citations into three methodological categories: empirical (data-based studies using quantitative, qualitative, or mixed methods), theoretical (focused on mobilized concepts, theories, or frameworks, and/or evidence-substantiated opinions), and review (explicit knowledge syntheses). Of these, 147 were empirical, 33 were theoretical, and 7 were reviews. Among the 147 empirical papers, 68 used qualitative methods, 64 used quantitative methods, and 15 used mixed methods. Finally, beyond the primary contextual findings, all papers were organized around four overarching thematic trends in the Canadian context. To foreground the knowledge synthesized in this work, Theme 1 is presented first, followed by key findings and subsequent themes.

### Theme 1: The convergence (and its extent) of religion with spirituality in relation to health

For some studies, spirituality was often expressed through “meaning-making” [1,2,3,4,5,6,7,8] and “sacramental recognition,” rather than an explicit language of religion. Interestingly, the term “sacrament,” which is a distinctly Christian term, appeared in multiple articles [9,10,11,12]. In particular, meaning-making was conceptualized by some scholars as a process of cognitive reappraisal salient to successful adaptation under chronic conditions or not easily alleviated by coping efforts [7,13]. Others relate finding meaning specifically to suffering and illness [14,15] and sometimes situationally mediated by music (as a “spiritual language”) [16]. Some scholars define meaning-making in relation to spirituality as simply making sense of one’s experiences [17]. Relatedly, spiritual distress occurs when one begins to question his or her meaning and purpose in life or feels hopeless [18]. Moral injury describes the profound distress that can arise from a violation of personal beliefs, increasingly recognized not only in military contexts but also in medical and other professional settings [19]. Spirituality is usually referenced apart from what articles call “highly institutionalized or religious form,” “organized religion,” dogma, or structure, where scholars advocate for the separation of spirituality from religion [1,20,21]. This distinction can even be seen in some surveys where participants were asked if they were spiritual, religious, both, or neither [22,23,24]. This distinction is evident in Indigenous communities, where spirituality holds a central role associated with traditional healing, and is often contrasted with Western and colonially imposed concepts of religion [25,26,27]. In another study, scholars differentiated between faith-based cognitive behavioral therapy and spiritual guidance concepts to show that spiritual concepts are not always strictly rooted in patients’ belief systems [28]. While there are many reasons for the contemporary trend of separating spirituality and religion, some scholars justify this from a public health perspective. Despite the relevance of spiritual health, they argue that barriers exist for embedding spirituality in public health because of its apparent—and often misconstrued—overlap with “institutional religion” in various contexts [29,30,31,7,32,33,34]. More specifically, scholars posit that the language and care practices surrounding spirituality can easily be perceived to be equated with institutionalized religion [7,35,36].

Instead, some scholars argue that spirituality is embodied and situationally conceived as an “orientation” [1,37], in which the individual’s spirit is expressed in everyday life via occupational performance in self-care, productivity, and leisure [38,20]. In other words, spirituality is an “everyday spirituality” [39]. Furthermore, some argue that spirituality is situationally marked by autonomy [40], emotionality [41], the individual’s spirit as a vital life force or transpersonal dimension [42,43], and independence from culture, politics, and geography [44]. This vital life force can either be interpreted as divine and theistic, characterized by the “care of the soul” [45], or as humanistic and secular [46,47]. Here, the discourse of “spiritual but not religious” is evident, in which spirituality is framed at the juncture of everyday life’s mundane and intimate aspects [1,48,45,49]. In other cases, scholars assert that there is no single definition of experience that can be named spirituality [50,51,52,53]. This has important implications for studies in which patients who did not identify as “spiritual” could still engage in meaningful conversations about what was important to them [54,10]. This was often because spiritual support involved activities like “meaning-making” and helping patients identify their value, rather than religious support, which included practices like prayer and attending to faith and belief systems [8]. Similarly, some studies do not directly address the relationship between spirituality and religion and instead view spirituality as a distinct concept encompassing connections in four domains: self, others, nature, and the transcendent, or a larger sense of mystery and meaning in life [55,56,6,57,58,47,59,60]. Other scholars described spirituality as a growing secular framework for ethical living, connectedness, and the search for the sacred [61,62,63].

Meanwhile, some papers discuss spirituality and religion as interrelated but still distinguishable [64,65,66,67]. Here, religion encompasses personal and institutional beliefs, institutional practices (like worship attendance), and social reinforcement [68]. Religion is generally presented as a structured system of beliefs and practices, often associated with specific institutions [54,69,70]. Spirituality, while possibly expressed within a religious tradition, is often described as a more personal, transcendental, existential, and privatized experience [71] and, situationally, is seen by researchers and laypeople as a more acceptable preference than “organized” religion [72,73]. For example, some scholars note that when distinctions are made between religion and spirituality, religion is the more likely construct of the pair to be neglected by mental health professionals [74]. Others argue that religion is only one form of spiritual expression [75]. In other cases, religion is considered a more orthodox form of spirituality [76].

Two papers collectively defined spirituality as including both publicized and privatized experiences [77,78]. They also noted that religion is operationalized as the system, while spirituality is the goal. One paper reported that spirituality and religion help people find meaning and purpose in life and include aspects of transcendence and transformation [67]. Another paper observed that the construct of religion is fairly well-grounded and easier to measure through attendance, while spirituality is much more porous and nebulous; nevertheless, the two necessarily interact [79,80]. Some papers denote that spirituality is more amorphous and often concerned with meaning, purpose, and transcendence beyond the individual [81,70,82]. While some articles acknowledge their interrelation, they also note that spirituality and religion “can inform each other but are separate” [81]. In other words, while religion often provides a framework for spirituality, individuals may experience spirituality outside of formal religious structures [64,50,69,83,66]. This framework is manifested, according to some scholars, as the phenomenon by which collective religious participation often precedes personal spiritual practices, cognitions, and virtues [84]. Other scholars further argue that while individuals may experience spirituality through religion, religious experiences and beliefs are not necessary for a healthy spirituality [85]. Therefore, people vary in their responses to what constitutes spirituality [50,52,53]. Other studies show the potential relevance of spiritual health across a variety of cultural contexts, particularly in relation to religious involvement [65].

In other cases, the distinction between spirituality and religion was made, but more implicitly [86,35,87,88,85,11]. For example, in studies that used survey methodology, many questions asked participants to rank the importance of their religious or spiritual beliefs in their way of life [89,90], or focused on religious or spiritual practices [89,91]. Similarly, other survey questions were written to distinguish spirituality from religion to clarify therapists’ perspectives [92], or referred to spirituality as pertaining more to existential well-being than religious well-being [93]. In another study in Canadian prisons, the author notes that prison staff often perceive Indigenous spirituality programs as religious [94]. In a study with Muslim Canadians, scholars note that while there are a variety of praying practices, religious prayer can increase the frequency of spiritual experiences [95]; another paper implicitly suggests that Islamic spiritual care and counselling is a religiously-based description of spiritual care and counselling [96]. Some scholars argued that secularized societies still uphold ritual and symbolic practices centering on experiences of the sacred, while not necessarily belonging to a religion or calling it spirituality. In other words, non-religious sources of spirituality are increasingly evident in modern cultures [97,98,99]. Through the (re)sacralization of society via new spiritualities, “believing without belonging” [100] has been used to describe the growing landscape of less organizational religious currents, such as “New Age” movements in Canada [101]. Conversely, the idea of “belonging without believing” is growing as well; that is, there may be communities that are “churched,” but not religious [100]. Thus, sacralization and secularization exist as prominent social swirling tides in Canada [100].

Among others, spirituality and religion were seen as intertwined, whereby one cannot have one without the other [102]. In the context of patient and hospice care, some spiritual care providers argued that they should not divorce spiritual care from their religious obligations; some argued that giving sufficient credence to the religious nature of their identity is as ontologically important as it is professionally important [103,104]. In another study, one Indigenous participant described their personal healing practice as inseparable from their Anglican and Indigenous beliefs [105]. Another paper argues that spirituality conveys both non-institutional spirituality and traditional religiosity [106]. However, this entwinement was seen primarily in the Afrocentric context in Canada. Here, spirituality is defined as the individual’s connection to a higher power or supreme being while equally requiring one’s commitment to an organized religious institution [107,108,109]. Building on this, some scholars also show that spirituality is expressed within religious sites [110]. Studies show that the Black Church as an “institutionalized religion” plays a significant historical and contemporary role in the spiritual lives of its believers [107] In some cases, religion and spirituality were even used interchangeably in African communities [111,112].

While some scholars argue that spirituality is a more popular expression today than religion—because many view religion as divisive and associated with war, conflict, and fanaticism—other scholars still define spirituality and religion synonymously or interchangeably to maintain clarity in research [113,114]. One paper notes that the interchangeable mention of religion and spirituality refers to, in either case, connections with an identifiable collectivity or system of faith or worship [115]. Another paper notes the usefulness of conceptualizing a single construct of both dimensions when doing quantitative research since there is evidence to suggest that, for many individuals, the “sacred search” often takes place within faith institutions [116,117]. In other cases, some studies use spirituality and religion interchangeably or together without denoting any justification or definition of the two [118,119,120,121,122,123,10]. Sometimes, a distinction between religion and spirituality was implicitly made on a conceptual level (or denoted with R/S terminology) with acknowledgment that religiosity and spirituality are complex [124,125,126,127,128,129,130,131,132]. However, some were inauspicious in sustaining that distinction [133,134,135]. For example, some studies mentioned the distinction but conflated religion and spirituality implicitly by failing to include a distinction in their quantitative methodology (e.g., measuring religiosity and spirituality by religious attendance, even when some individuals who call themselves spiritual may not attend events of an “institutional religion”) [133,135,136,137]. More specifically, other scholars acknowledged the limitations of religion/spirituality in quantitative methodology and their inability to tease apart that construct [101]. Other scholars noted that there is no easy, logically consistent way to reconcile the incongruence between “R/S beliefs and behaviours and “R/S identities” [131,138]. Generally, in quantitative studies, researchers assess a person’s spiritual or religious “levels” with one of three indicators: attendance, religiosity, and religious affiliation [129].

### Key findings

For this paper, we present only the key findings from our top primary contexts.

#### Youth and adolescent health.

Studies [1,65,55,29,139,56,135,140,116,58,114,47,141,44,85,39,142,71,60,33] found that higher perceptions of spiritual health’s importance, including its sub-domains (connections with self, others, nature, and the transcendent), were linked to positive health outcomes, lower psychological and somatic symptoms, and reduced likelihood of cigarette smoking, alcohol use, marijuana use, and sexual intercourse [65,55,29,139,58,33]. Children with religious involvement reported higher self-rated importance of spiritual health compared with non-involved peers [141]. Scholars also acknowledged growing mental health concerns among Canadian youth; they suggested that spiritual health—particularly a connection to nature—might help alleviate this crisis [55]. Relatedly, scholars suggested that promoting healthy connections with others, nature, or the transcendent can indirectly improve mental health by strengthening connections to self – these were considered “spiritual themes” because there was some general agreement that spiritual health involves a “way of being” and deeply felt human connections to others, nature, or the transcendent [56,58]. Furthermore, among Indigenous youth, spirituality supported a positive sense of self and community reintegration [1]. Among Black youth, religion stigmatized mental illness but was still seen as essential in mental health support [140].

#### Psychiatry and psychiatric disorders.

In this paragraph, the terms “religiousness” and “spirituality” are used as they appeared in the original studies. In many papers, these concepts were conflated or used interchangeably; the wording here reflects the original usage to faithfully represent the findings. Studies [64,113,91,83,22,119,120,133,2,79,143,80,117,144,93,28,127,82] note a distinction between harmful religious delusions and healthy religious beliefs in individuals with psychotic disorders [113]. While delusions can worsen one’s mental health, healthy religious beliefs and practices often stabilize patients; this is substantiated by the fact that 71% of patients in one study use religion as a tool for hope, purpose, and meaning [113,127]. Relatedly, mental health professionals face challenges in distinguishing spiritual experiences from psychosis and setting boundaries in spirituality discussions [82]. Another paper found that individuals who place higher importance on spiritual values—defined as meaning, inner strength, and understanding—are more likely to experience mood and anxiety disorders [64], a connection that may reflect active seeking of those spiritual values as a coping mechanism [64]. Psychiatrists were encouraged to consider religion and spirituality in patient’s treatment journeys and psychiatric curricula [64,83,144]. Another study showed that religious/spiritual individuals had significantly lower levels of social anxiety and lower levels of depression severity [91]. In other papers, interestingly, a stronger self-identification with spirituality or religiousness was associated with higher levels of depressive symptoms [119], while frequent attendance at worship services is linked to lower levels of depressive symptoms [119,120,2]. Two papers discussed that psychiatrists’ beliefs and practices predict inquiry into their patients’ spirituality [22,133]. Similarly, patients in one study vocalized that spirituality is an important issue to address as part of their psychiatric treatment [22]. Psychiatrists in the Christian Medical and Dental Society considered incorporating prayer and other Christian practices for their Christian patients as part of treatment [133]. Supportive faith communities are considered as crucial as individual spirituality in influencing mental well-being [79]. Meanwhile, a Spirituality Teaching Program significantly reduced depression severity and increased response and remission rates [28]. Two studies showed that men and younger women with low spirituality experienced higher levels of body shame, which increased eating disorder symptomatology [143,80].

#### Occupational therapy.

Studies [50,145,38,70,146,20,4,46,92,51,147,148,75,63] discussed the integral role of spirituality in occupational therapy and the need for therapists to be sensitive to their clients’ spiritual dimensions [50,146,20]. The Canadian Association of Occupational Therapists has upheld this ontological tenet—that there is a spiritual dimension to health—since the late 1990s [145,4], yet many occupational therapists are unsure how to implement spirituality into practice, particularly regarding boundaries and ethical issues [92,147,63]. For example, while prayer may deepen the client-therapist relationship and is noted in occupational therapy as a spiritual modality, it raises concerns such as perceived coercion, role confusion, the therapist’s possible lack of familiarity or comfort with its use, and the employer’s unease with the formal use of prayer in therapy [46]. Scholars also note that spirituality discussions in occupational therapy have been hindered by the lack of a common definition [145,51], and that spiritual language, discourse, and praxis must be considered since they are socially constructed phenomena [148].

Meanwhile, narrative approaches have the potential to explore spirituality, especially in mental health [38]. In particular, spiritual enrichment may occur through narrative as one frames and reframes actions and events, retrospectively revises, selects, and orders past details to justify and find purpose in one’s current life situation [38]. However, approaching spirituality primarily through a mental health lens risks addressing it only indirectly. Other scholars built on this and argued that an assessment of spirituality within occupational therapy should be ongoing, since spiritual assessments may evolve throughout rehabilitative and re-integrative processes [20].

#### Indigenous communities.

Studies [149,105,150,151,152,48,25,26,32,27,11] discussed the importance of spirituality for Indigenous identity and across their life histories, especially since the pandemic [149,152,27]. One paper highlighted spiritual aspects of health as key to connection with Métis ancestry [152]. Former Indian Residential School students shared that spirituality, cultural practices, or “forgetting” were necessary for healing [105]. Another study found that spirituality has an impact on sexual decision-making at the individual level [150]. Relatedly, scholars note that Indigenous spiritual practices and language preservation are closely interrelated and broader than current perspectives on biomedicine [151,48,25], with their revitalization shown to improve individual and community well-being, particularly in reducing youth suicide rates [151]. Because of this impact, some scholars have advocated for greater integration of spiritual health into current health programming [32]. Furthermore, words and phrases such as “healing” and “living in harmony with nature and in community” were associated with health [105,25,26,11]. Finally, the resurgence of Indigenous cultural and spiritual traditions was noted to be a type of “decolonizing spiritual practice” [27].

#### Patient care.

Studies [54,81,42,103,35,136,125,14,59] discussed the value of spiritual history taking. Using the FICA Spiritual History Tool helped healthcare providers understand patients’ spiritual needs and led to more person-centred care [54]. One article notes Gadow’s three-level ethical framework, which includes a relational narrative approach as a helpful tool for nurses to deepen their understanding of patients’ spiritual beliefs [14]. Understanding patients’ spiritual needs resulted in improved communication and trust between healthcare providers [54,103] and increased satisfaction with healthcare services [103]. This is also evidenced by positive outcomes in spiritual group interventions [125] or in the claiming of spaces for sacred purposes within healthcare settings [59]. Scholars also found physicians often reluctant to discuss spirituality [81] and more likely to ask about beliefs only if they deemed it important [136]. In another paper, both patients and healthcare providers advocated for addressing basic spiritual issues as the responsibility of an interdisciplinary team while recognizing the need for specialized and embedded support from a spiritual care professional [35].

#### Spiritual care providers/spiritual health practitioners/chaplains.

Studies discussed that spiritual care, while integral to healthcare delivery, is particularly effective in helping employees cope with stress in traumatic or grief-filled environments and in providing multifaith care for patients [104,86,96,153,154,36,9,45,155]. Relatedly, spiritual health practitioners offer multifaith, multicultural care for patients and employees, though their integration often depends on colleagues’ attitudes toward religion and spirituality [9]. Muslim spiritual caregivers, for instance, use traditional healing techniques but require theological approaches sensitive to both Shia and Sunni traditions [96,153]. Furthermore, one study found that spiritual health practitioners mitigated compassion fatigue before and during the COVID-19 pandemic [86]. Another paper reviewed the strengths and challenges of evidence-based spiritual care practice, cautioning spiritual care providers not to overemphasize research at the expense of diminishing the transformative power of presence and the clinician’s safe and effective use of the self [45]. Additionally, implementing evidence-based spiritual care practices presents challenges, particularly in research literacy [45]. Other papers noted that chaplains face difficulties securing funding, necessitating strategic positioning with public funders, and some work part-time to prevent burnout [86,36]. Finally, one paper shared that the language of “spiritual and religious” should be included in the spiritual history assessment by spiritual care providers [104].

#### Palliative and end of life care.

Studies [8,76,23,156,157,123,16,67] discussed, for example, that implementing the Comfort Measures Order Set, which included an automatic referral to the Spiritual Care Team, led to a notable increase in spiritual care consultations for patients receiving end-of-life care [8]. This study also highlighted the need for standardized documentation practices by the Spiritual Care Team to better capture the nature and impact of their work and make it more visible to other healthcare professionals and administrators [8]. Another paper argued the importance of assessing the patient’s spiritual coping and spiritual problem-solving style in end-of-life care [76] and, by extension, to appreciate the spiritual dimension in patients’ lives [67]. Relatedly, one paper mentioned spiritual distress as a common theme felt by family members of ICU patients and that clinical strategies for addressing spiritual distress are needed [23]. One paper discussed that palliative care catalyzed team members’ spiritual journeys [156]. Another study identified five spiritually integral bedside skills as essential to spiritual care – hearing, sight, speech, touch, and presence [157]. The integration of these bedside skills with the intrinsic qualities of healthcare professionals, including their values and spiritual beliefs, was essential to their application in spiritual care [157]. Finally, other papers augmented the need for professional development in palliative and bereavement care [123] and explored the theological use of music in spiritual care [16].

#### African and/or African-Caribbean communities.

Studies [107,110,111,108,112,109,158] found that spirituality and participation in African church communities served as a source of strength, a coping mechanism for racism and discrimination, and a vital component of health and well-being. Another study discussed that religious spaces of worship for African immigrants were associated with physical, social, symbolic, spiritual, and emotional well-being [110]. In one study based in Nova Scotia, several women claimed that their spiritual health was as important and significant as their physical and mental health [107]. Relatedly, African Nova Scotians shared daily experiences of spiritual alienation—a disconnection from non-material and morally affirming values—because of racism and discrimination [107]. Other studies mentioned that spiritually-related occupations helped African women reinterpret racism and navigate challenges [108,112], while spirituality reduced depressive symptoms in African Canadian women with HIV by fostering psycho-spiritual coping strategies and a belief in spiritual protection [111]. One paper advocated for healthcare providers to be aware of the spiritual needs of families of African descent and to include spirituality as part of end-of-life care planning. Finally, African-Caribbean healers’ reflections on collaboration and networking with Western healthcare practitioners uncovered various obstacles, barriers, skepticism, and perceived illegitimacy of their specific faiths, cultural beliefs, and healing practices [158].

### Theme 2: The dimensions of health which spirituality/spiritual health is often conceptualized against or discussed with

Spirituality was often conceptualized against or discussed with mental health [1,65,40,145,69,38,29,139,94,95,111,83,22,119,120,133,2,108,112,109,159,79,143,160,161,149,134,121,105,162,3,97,77,163,164,135,140,151,165,7,13,116,117,57,166,167,58,25,168,122,90,169,96,154,106,98,114,101,93,47,170,125,44,85,39,9,102,171,142,28,26,126,27,60,172,19,66,130,132,123,10,173,138,63,24,84,49,67,174,12,74,155,175], with two papers specifically mentioning cognitive health [122,85]. These papers focused on a range of aspects, from alleviating depressive or post-traumatic symptoms and racism-related stress to equipping people with healthier coping strategies, therapeutic treatments and hope. Other papers focused on rumination, internalizing/externalizing emotions and behaviours. In other cases, spirituality was discussed in relation to overall health and well-being [64,40,89,145,107,37,69,38,70,146,20,68,118,160,30,31,4,5,46,6,176,13,152,17,43,147,169,96,177,88,78,98,124,101,141,158,44,178,179,14,100,62,59,26,71,156,157,148,45,180,33,181,63]., covering topics such as disabilities, illness, and impairment. In other studies, spirituality was connected to emotional health [64,40,145,107,144,136,52,99,124,157,42,82,16,94,175], social and psychosocial health [64,54,107,38,42,20,110,95,120,162,140,116,90,147,182,99,72,102,27,75,15], or physical health [64,40,145,69,20,143,80,162,183,150,13,57,58,99,137,21,129,84]. From these citations, only one paper focused on reproductive health [150]. Only two papers specifically referenced public health [183,36], and one paper focused on existential health [107].

In other cases, spirituality was a dimension of health and often mentioned as the “fourth domain of health alongside physical, mental, and emotional dimensions; in some papers, this was viewed as “holistic health and well-being” [54,55,56,41,104,48,154,184,23,32,73,18,127,10,82,34,11,155]. In other cases, papers mentioned spirituality as both a dimension and determinant by nature of its interrelatedness with the other dimensions of health [50,56,146,103,159,46,92,51,35,78,47,61,185]. Spirituality was seen as an essential element of human functioning that can be impacted by illness, injury, or disability. Conversely, it can also be a source of strength and resilience in coping with health challenges. In a similar vein, one study used a mental health indicator based on the frequency of psychosomatic symptoms, and the findings demonstrated a link between stronger spiritual connections and positive mental health [56].

### Theme 3: The presence and role of interfaith dialogue

The included papers addressed various aspects of interfaith dialogue and collaboration, though the depth and focus of their discussions varied. While some advocated for openness to diverse spiritual symbols and traditions and questioned whether spirituality should be included in practice, they did not explicitly delve into interfaith dialogue [70,127,172,63,15,11]. Another paper emphasized the relational strength of religious sociability as central to successful interfaith collaborations [72]. In contrast, others refrained from exploring relationships between different religious groups but raised critical interfaith and theological questions, such as the intersection of spirituality and evidence-based studies. One paper, for example, highlighted that evidence-based spiritual care assumes empirical evidence can demonstrate the effects of spiritual care on patients, urging that such inquiries should take the form of an ecumenical and interdisciplinary dialogue that respects diverse experiences [42].

Meanwhile, the role of interfaith collaboration emerged in discussions concerning spiritual care providers, spiritual health practitioners, and professional chaplains [103,7,96,153,87,9,59,186,10,155]. Professional chaplains were noted as facilitators of interfaith collaboration in healthcare who complemented local religious leaders while addressing specific needs in medical settings [103]. Despite the availability of multi-faith chaplaincy programs in Canadian hospitals, patients often received less spiritual support than in home care, where spiritual community members may have offered more consistent visits [186,10]. Spiritual care practitioners often navigated “hybrid spaces” where deep connections transcend religious differences, and fostered unity while maintaining individual beliefs [9]. This is paralleled in hospitals where “third spaces” have been created—liminal areas embedding symbols and artifacts from multiple faiths to blur traditional boundaries [59]. Additionally, other papers discussed therapists’ strategies for integrating clients’ faith into practice [92]. Using prayer as a bridge across traditions [7] and delivering multi-faith spiritual care without compromising caregivers’ own beliefs [187,153,87] were also highlighted.

The growing need for familiarity with diverse faith traditions due to increasing religious diversity, particularly in Long-Term Care Homes as Canada’s population ages, also emerged as a key consideration [88]. One study highlighted the variance among Christian traditions, which complicates efforts to understand how faith communities support older adults experiencing depression [79]. In some contexts, challenges to interfaith collaboration were noted, such as offenders in prisons rejecting Indigenous spirituality programs due to faith-based tensions [94]. Similarly, studies involving participants from various faith backgrounds—Muslims, Christians, Indigenous individuals, and those identifying as Atheist, Agnostic, or Buddhist—offered insight into interfaith dynamics but also underscored the complexities of such engagements [110,161,45,129,130,180,33,173,15,175].

Christian-Indigenous interfaith dialogues were explored in several studies [105,150,152,25,26,138,174], including the Anglican Church’s engagement with Indigenous spirituality [105]. Additionally, a study highlighted spiritual leaders from diverse faiths [183], and some papers noted shared values and practices across different traditions [77,46,47,178]. However, the challenges of building connections across worldviews were also acknowledged [187]. Scholars called for psychiatry residency training to include Christianity, Islam, Judaism, and First Nations spirituality to better address religious diversity in mental health care [144]. One paper delved into metaphysical interfaith dialogues, arguing that Christian metaphysics is essential for spiritual health in the West, while others debated revisiting Christianity without its metaphysical core [36].

### Theme 4: Cultural considerations in relation to spirituality and health

While some papers did not go into detail about cultural considerations, a broad swath of papers stressed the importance of providing culturally sensitive care, given the multicultural context in Canada [8,103,22]. Some explored immigrant cultural perspectives on spiritual and religious beliefs [110], noting that multiculturalism may contribute to the increase in the number of people who identify themselves as spiritual but not religious [83]. For Indigenous communities, cultural traditions and ceremonies were central to spiritual well-being, which embodied their “cultural spirituality” [1,94]. For African-heritage peoples, the Black Church has historically and presently played a significant role in shaping spiritual and moral frameworks in African Nova Scotian communities and has been a source of strength, survival, transformation, and transgression [111,108,112,109]. Scholars also advocated for the integration of both in Western society since African humanism, as some papers stressed, does not separate God from humanity [107,37].

Meanwhile, the link between religiosity and subjective well-being, however, varies by cultural and social integration contexts. The culture-person congruence thesis posits that happiness increases when personal traits align with societal values [89]. Furthermore, one’s spiritual and religious involvement impacts mental health and can vary based on cultural context and social integration within the religious community [113]. Some scholars broadly examined how Western cultural assumptions and historical shifts have shaped the understanding and practice of spirituality within a secular (and post-secular) society [70]. Other papers discussed the idea that health inequalities may be linked to what scholars define as a third sub-domain of spiritual health, namely a disconnect and anonymity from nature caused by a lack of intentional exposure to the natural environments [55,56]. Other cultural factors threatening children’s spiritual health include the fast pace, high demands of modern life and consumerism [55]. Others noted that there may be some cross-national and gender differences in the extent to which health domains (including spirituality) impact young people [29]. Additionally, as mentioned earlier, the culture around the Canadian Armed Forces operates under a unique “unlimited liability” rule, whereby members may face extreme risks and responsibilities that other Canadian employees do not [159]. This context can lead CAF members, or their allies, to carry out actions on behalf of a democratically elected government that may be deemed immoral or morally troubling [159]. Witnessed or perpetrated activities can cause moral or spiritual wounds, including a deep sense of guilt or shame [159].

In one study, spiritual care providers in an increasingly pluralistic context navigated diverse faith traditions while offering care to patients across a wide spectrum of beliefs. All paid spiritual care providers had graduate-level education, including Clinical Pastoral Education (CPE) training. Scholars noted that the CPE model embodies a form of secularized spirituality designed to address any belief system, making it particularly suitable for spiritual care within healthcare institutions [9]. Lee (2002) referred to this as a “legitimizing strategy,” marked by a shift in terminology from religiously affiliated chaplains to professional spiritual care providers. [l in S2 File] The Canadian Association for Spiritual Care/Association canadienne de soins spirituels (CASC/ACSS) now refers to CPE as Clinical Psychospiritual Education, which reflects this broader shift as well. [d in S2 File]

Finally, the literature identified two cultural trends: privatization and de-privatization. Scholars noted that the shift towards scientific paradigms has privatized spiritual and religious symbols, and by extension, diminished their public significance and authority; this privatization has impacted the understanding of the mind-body-spirit connection, with a historical shift from public care of souls to a focus on bodily health [70]. Some suggested that the dominance of scientific paradigms in Western culture has led to a focus on measurable aspects of health, which further privatized spirituality [20]. Conversely, scholars observed a de-privatization or the public sphere’s re-enchantment with religion, evidenced in the increasingly growing religious groups participating in public policy and welfare services provision, as well as the incorporation of subjective well-being spiritualities into broader cultural practices [59].

## Discussion

Through the process of reviewing the included 187 citations, this scoping review has not only identified primary health contexts in which spiritual health and spirituality (in relation to health) is discussed, but this review also describes key thematic trends in Canada across all these studies. Showing this level of breadth and depth in a scoping review, especially in a review that covers 187 articles, is part our methodological novelty, which is not usually common in scoping reviews. At first glance, readers of this article might feel overwhelmed by the “thick culture” of it all. However, perhaps it is this texture that necessitates and illuminates the complexity of this topic.

When examining the primary health contexts, there are variegated disciplinary or occupational tendencies in approaching spiritual health. Let us, as an example, focus on psychiatry since this primary context augments the complexity of this topic. Within psychiatry, numerous cross-sectional studies have examined the relationship between religion/spirituality (R/S) and psychiatric disorders in relation to mental health. However, a common limitation of these studies is their inability to establish causation, despite the temptation to do so. Relatedly, psychiatric literature seems to struggle to engage with spirituality and spiritual health, both within psychiatric practice and as phenomena in their own right. For instance, the association between the frequency of prayer and higher levels of psychiatric or mental health issues could either reflect a response to existing distress or be a contributing factor to the distress itself. Similarly, lower religious attendance might either result from mental health challenges, such as amotivation or isolation, or act as a contributing cause of distress in individuals who might otherwise have attended religious services. In the context of quantitative studies in psychiatry, longitudinal studies offer a more robust methodology for exploring these relationships, as they can provide evidence of causality over time. This also shows how the literature views the psychiatric effects of religious/spiritual involvement and conceptualizes spirituality and spiritual health in themselves. Additionally, the focus areas of Canadian studies on R/S also reflect the specific interests of researchers in this field. Consequently, these studies may not comprehensively represent the broader “health” spectrum, particularly in terms of spiritual health and well-being. Moreover, spiritual health and well-being are sometimes treated as tautological concepts in surveys, with overlapping dimensions being measured. Finally, many studies in this field have been conducted over the past two to three decades, a period marked by significant generational and cultural changes in Canada, including immigration, increasing secularization, and now post-secularization (which will be discussed below).

Secondly, while adolescent health is the dominant primary health context in Canada, the examination of spiritual health and spirituality in aging communities is relatively underexplored in the included articles. This statement is not meant to diminish the spiritual care programs that already exist in Canadian long-term care homes. Rather, it acknowledges that discussions of spiritual health are less visible in the peer-reviewed literature, even though they may appear in other venues, such as books, which were outside the scope of this review. For example, Simon Lasair’s (2025) recent book on spiritual health addresses spiritual care programs in long-term care homes (p.3). [k in S2 File] Aging communities offer a good entry point into this topic because of the aforementioned generational shifts. For aging communities, it can be inferred that many of these populations will come from more “organized” religious traditions than their successors. This raises important issues, namely within healthcare pastures of thought and praxis. For example, how can health professionals be informed and educated about religious traditions? Should faith-based collaboration between health and religious sectors be judiciously increased to better meet the needs of older adults? Therefore, future research focusing on life history, intergenerational dynamics, and older adult perspectives may be particularly fruitful in the Canadian context.

Meanwhile, when examining key thematic trends in this review, a notable aspect of this evolving discourse is the increasing openness to discussions surrounding the spiritual dimension of health. However, the lack of a clear, universally accepted definition presents a significant barrier to fostering constructive dialogue. At the same time, the possibility of achieving such a definition is uncertain, as spirituality is inherently characterized by its deeply contextual nature. As seen with other scholars, many leaders in spiritual health propose “working definitions” but remain hesitant to endorse a single definition because of the complex, fluid, and elusive, contextual nature of spirituality. [a, j, v (in S2 File), [136] These working definitions are useful for dialogue and debate but are not considered the “ultimate definition” of spirituality. Furthermore, the work that Puchalski et al. (2009) has done through consensus conferences has made a significant impact on the American and international research literature, but this scoping review suggests that there is a lack of consensus in Canada. [u in S2 File] This discursive undulation creates an ongoing tension between the impulse to define spirituality and the recognition that its very essence may resist precise definitions, which raises questions about the practicality and utility of attempting to do so. Ultimately, this foregrounds how challenging it is even to describe these phenomena, and therefore makes it even more difficult to engage them clinically. This may also explain why, for instance, efforts to integrate spirituality into public health frameworks are sometimes approached in a limited or perfunctory manner, or why there is a larger body of literature on culturally competent care in health curricula and patient care protocols than on spiritually competent care. Overall, the dangers of this ambiguity may mean that spirituality is overlooked as a resource for supporting people in their lives, or dismissed as inappropriate for the public or policy sphere because of its implied or assumed (even incorrectly) connection to religion—something a clear definition could clarify. However, the strengths of not advocating for a clear universal definition of spirituality could arguably outweigh these limitations as well. Spirituality is arguably inherently contextual and expressed differently by people across time and place. Thus, one can argue that it needs to be reflective of and respond to the needs of the people who are using the word in a specific context. Therefore, while spirituality can be described, any claim to solely a final definition of spirituality would potentially risk alienating anyone who is not reflected in the specific definition that was proposed.

Contrary to the arguably popular belief that certain religions—and, more specifically, religious rituals—have become vestigial, religion still emerged as a prominent force in the Canadian context, despite the deliberate omission of this word in our search strategy. This was broadly evident in the converging discourse between spirituality and religion and seen, for example, in African/African-Caribbean communities across Canada as they navigate through grief, racism and oppression. While the relationship between spirituality and religion, as evidenced in this review, is “thick” and “sticky,” communities in Canada also demonstrate that, in certain contexts, spirituality is not diametrically opposed to “organized” religion, and both can be critical, mutually buttressing one another in the life of the community.

Furthermore, the use of the term “sacrament” within literature discursively casts a vivid light on a notable terminological and conceptual complexity. While *sacrament* is distinctly a Christian theological concept, its appearance in broader discussions of spirituality points to a possible syncretism between religious and spiritual frameworks. This raises important questions about how language rooted in specific religious traditions is repurposed in multi-cultural and post-secular contexts. The invocation of sacramental language reflects an effort to convey deeply meaningful and communicable aspects of human experience, yet this linguistic borrowing also highlights the challenges of disentangling spiritual practices from their religious origins. The conceptual tension demonstrates how spirituality may draw upon religious symbols and language to articulate universal human experiences, even as it seeks to differentiate itself from organized religion. In this way, it challenges the lingering tendency to strictly delineate spirituality from religion.

Yet, while the undercurrents we see, such as the example above, may be attributed to religious syncretism because of an increasingly multicultural and post-secularist society in Canada, it is arguably not the full picture. [s in S2 File] Using another interpretive lens, Canada’s undercurrents may loosely mirror the enchantment of German Romantic poets that Charles Taylor (2024) has meticulously fleshed out in his most recent book, *Cosmic Connections*. [w in S2 File] In his book, Taylor argues that the Romantics recover a language of insight through poetry and works of art to pull back the veil on otherwise hidden connections between humans and the cosmos. With this language, the Romantics are also invoking a non-religious transcendence in their work. Here, language becomes more than “an instrument to encode information” and instead, higher and more creative, to “transform [their] relation to the situation it figures for [them]” (p.18). Furthermore, Taylor describes the Romantics as “rebelling against mind-body dualism” (p.5) and longing “for a unification of self, unity with [their] emotions, with nature in [them], and with nature as a whole” (p.5), whereby freedom is redefined as “full self-realization” (p.6). Similarly, based on the aforementioned results discussing spirituality, we are situationally met with this growing transcendentally flavoured language. To borrow Taylor’s words, this language is characterized by an “an unending series of subtler languages” (p.38) that “invoke entities whose ontic status is not clear” (p.61). While this cannot be said for all of our results as some language in these articles reflects coherent ontically grounded accounts in that they draw from certain traditions of theology and philosophy, this suggests that the verve of spirituality in Canada is not monolithic but variegated.

While the present era and the Romantic era are different, both suffer from the dark undertows of disenchantment (this exact word is also articulated in our findings) [142,11]. In the Romantic era, this loss finds its genesis in “the advance of certain technologies… and the resulting speed of travel and communication” (p.23). Other factors include instrumental reason as well as mind-body dualism. [w in S2 File] Similarly, the loss experienced in Canada may inspire a resurgence of romanticism, as both the challenges posed by the Romantics and current spiritual impulses are deeply shaped by these conditions. The degree of this “romantic” tendency varies across communities. For Indigenous communities, for example, the beautiful focus on nature is seen as an inherent continuity of their ancestors’ enchantment. Relatedly, in other studies, spiritual health is defined to have a sub-domain that connects oneself to nature. Thus, culturally, we are beginning to embody some of the values that the Romantics sought to pursue. [m, s in S2 File]

Yet, the corresponding aspiration to recover from this loss remains subordinate to the broader Western paradigm in Canada, especially in our discussion of health. In an increasingly medico-scientific society, in the words of Taylor, “metaphor has to be used sparingly or not at all” (p.19). Furthermore, subtler languages of spirituality and religion may resist “newly publicly established references” (p.38) and arise from “personal experience which may be hard to communicate to others” (p.48). This disconnect punctuates why healthcare efforts to describe spirituality can be so challenging, as these attempts often lack the conceptual tools to understand religious language or experiences of the transcendent. Thus, this is both a conceptual and existential problem.

Finally, in examining the broader discourse of these articles, we see that there is less articulation of how the body knows and senses spirituality and religion in relation to health. While spirituality is situationally discussed in terms of transcendence, the focus on people’s sensory experiences is often lacking. While it would be logically fallacious, given only this data, to conclude that this phenomenon indicates a lack or demise in people’s ability to sensorily attune themselves, what we can modestly infer is that, at the least, philosophically, Descartes’ legacy of mind-body dualism in the Canadian context presupposes the mind as the source of the self, rendering the body less relevant. Here, we observe a reimagined form of Gnosticism (a second-century religious movement that was considered heretical by the Early Church which saw the body as a prison in which the soul was trapped) in Canada, whereby the materiality of the body is negated while concomitantly transformed into a commodity that can be manipulated for various purposes. [g in S2 File] This is why the body, situationally, can be changed—not because it is irrelevant, but because the liberation of the self residing in the mind positions the body as secondary. This idea is also reverberated in Charles Taylor’s (2018) *The Ethics of Authenticity*. [x in S2 File]

## Practical applications

With this scoping review as a point of departure, the question becomes: what degree of granularity is required for meaningful practical application? In what follows, we propose practical applications at the clinical and public health levels, while acknowledging some challenges.

One application of this work is the actualization of “social prescribing” in current public health and clinical practice across Canada. Social prescribing is “a means for trusted individuals in clinical and community settings to identify that a person has nonmedical, health-related social needs and to subsequently connect them to nonclinical supports and services within the community by coproducing a social prescription—a non-medical prescription— to improve health and wellbeing and to strengthen community connections” (p. 9). [o in S2 File] Valerie Michaelson, who is a co-author of this scoping review, is already advocating for social prescribing in the context of meaning and purpose in life among adolescents. [r in S2 File] Following Pickett et al. (2025) and Muhl et al. (2023), we propose that social prescribing can also be extended to support spiritual health. [o, r in S2 File] The breadth and depth covered in this scoping review can serve as a comprehensive guide to the types of activities, communities, or practices that may foster spiritual well-being. In this way, social prescribing offers a practical mechanism for acknowledging spirituality as a legitimate determinant and dimension of health, and for creating informed pathways that support individuals in cultivating the forms of meaning and connection most resonant with their own spiritual lives. Furthermore, social prescribing implicitly resists the growing medicalization of health while also, as Muhl et al. (2025) argue, ameliorating pressure on healthcare services and lowering healthcare costs. [n in S2 File] Because of its potential to contribute to broader health system reform, this approach has now been adopted in more than 30 countries, with its use continuing to expand. [n in S2 File]

Meanwhile, drawing on the included literature in our scoping review from psychiatry, patient care, and beyond, there are already a few practical implications that are worth emphasizing further. First, as Marilyn Baetz and colleagues note, it’s important to address the spiritual aspect of patients’ lives —be it positive, negative, or neutral to care for the whole person; as an extension, the involvement of spiritual advisors, such as clergy or chaplains, is important—though this remains dependent on provincial regulations and funding structures governing Spiritual Care Practitioners and related roles [83]. Second, as mentioned before, the integration of spiritual history-taking into routine clinical practice is noted in the literature. Relatedly, several spiritual assessment tools [54,66]—including the FICA tool (Faith, Importance/Influence, Community, and how these issues should be Addressed in care), first proposed by Puchalski (2014) at the National Consensus Conference on palliative care—are identified as concrete strategies for clinicians. [t in S2 File] We suggest that broader uptake of these tools can offer a practical pathway for integrating spirituality into assessment and care planning across health settings.

Following Puchalski (2014), we reinforce the following key principles for taking a spiritual history in clinical and public health contexts. [t in S2 File]

- Recognize spirituality as a potentially significant component of every patient’s life and well-being.
- Address spirituality at new visits, annual examinations, and follow-up appointments when appropriate.
- Respect each patient’s privacy regarding spiritual beliefs and practices.
- Maintain awareness of your own beliefs and avoid imposing them on patients.
- Refer to chaplains, spiritual directors, or relevant community resources when needed.

Another possible approach is convening a national consensus conference, bringing together experts (including Valerie Michaelson, an author of this review, who has done extensive work defining spiritual health) from multiple disciplines to work toward a shared understanding of spirituality in clinical practice. Puchalski’s conferences, as we alluded to, were effective in establishing a broad conceptual understanding of spirituality, though these definitions have still been criticized for not being sufficiently connected to clinical practice. Walton (2012) articulates some of these critiques from a chaplain’s perspective, [z in S2 File] while Lasair (2025) engages them more fully and constructively in his book on spiritual health. [k in S2 File] If the goal of our large-scale review is to capture the full complexity of this vast yet important topic, the question then becomes: how can we achieve clarity and consensus? A consensus-seeking conference may offer one viable path forward. Yet, we also recognize that the thick and textured elements of spirituality in the Canadian context are important for policymakers to understand, so that policies can be crafted in ways that are attentive to difference, plurality, and lived spiritual realities, rather than relying on overly generalized notions of inclusion. As authors, we recognize that in the current American landscape on spirituality, clarity does emerge from the research literature, but its applicability to the Canadian context is uncertain due to differences in religiosity and spiritual expression. Moreover, several challenges identified in the Canadian literature also persist in the American literature, suggesting that policy recommendations cannot be based on the existing literature alone. An interdisciplinary white paper, drawing on experts to build consensus around a Canadian clinical approach to spirituality, may be another practical step forward.

There are also some methodological considerations to account for, in that each of the researchers included in our scoping review is approaching the question from within their own professional context and competence. Given the significant differences among the professional competencies and cultures represented in the paper, consensus might be very difficult to reach, particularly since the goals of care for each profession might differ considerably. This raises the question of whether taking a patient-oriented approach might be part of the next steps. Although research increasingly shows that practices such as prayer and meditation can support health and healing, what remains missing in many care settings is an awareness of what is actually familiar or meaningful to the person receiving care, especially amid growing religious and spiritual diversity. How do patients understand their spiritual concerns? Might it be possible to build a grounded theory of spirituality based on qualitative interviews with patients? To our knowledge, there are very few studies of this kind, particularly in Canada, so taking such an approach might help us render greater clarity while also ironing out some of the challenging methodological and conceptual problems revealed by our paper.

## Conclusion

In summary, assembled here for the first time, the knowledge synthesis and thematic analysis of this literature provide the scaffolding for this paper, while offering a critical and rich window into the world of spiritual health and spirituality (as they pertain to health) in the Canadian context. In examining 187 articles and illuminating primary health contexts in which spiritual health/spirituality is discussed in Canada, our findings highlight where future research on this topic may be directed. Importantly, this review also identifies key thematic trends, namely the convergence (and the extent of it) of spirituality with religion in relation to health; the other dimensions of health that spirituality is connected to or discussed with; the presence and role of interfaith dialogue; and the cultural considerations in relation to spirituality and health. Overall, by moving beyond the descriptive content of our included articles and intentionally mapping out the discursive content as well, this review has not only filled critical gaps in Canada’s landscape of spiritual health, but also critically shows that this spiritual milieu is one that is “thick” and “textured.” Yet, this complexity is a critical thoroughfare to deeper understanding.

## Supporting information

S1 Table Prisma-Scr Checklist Table. (PDF)

S2 Table Search Strategy Across All Databases. (DOCX)

S3 Fig A Visual Landscape of Spiritual Health and Spirituality in Canada. (PDF)

S1 File Full Table of Included Publications. (XLSX)

S2 File Reference list. (DOCX)
