# Correction: A scoping review and thematic analysis of the landscape of spiritual health and spirituality in Canada

**DOI:** 10.1371/journal.pone.0354416

**Published:** 2026-07-21

**Authors:** The PLOS One Staff

## Notice of Republication

This article was republished on July 09, 2026, to correct in-text citation errors that were introduced during the typesetting process. In the “Theme 2” subsection of the Results section, there was an error in the last sentence of the first paragraph. It originally read “Only three papers specifically referenced public health [183,36], and one paper focused on existential health [107].” This was changed to read: “Only two papers specifically referenced public health [183,36], and one paper focused on existential health [107].” The publisher apologizes for these errors. Please download this article again to view the correct version.

## Supporting information

S1 File Originally published uncorrected article. (PDF)

S2 File Republished corrected article. (PDF)
