# Supplementary material for: A scoping review and thematic analysis of the landscape of spiritual health and spirituality in Canada
Source: PLoS One. 2026 Feb 20;21(2):e0340854. doi: 10.1371/journal.pone.0340854 (PMC12923021; doi:10.1371/journal.pone.0340854)
Supplement: S2 Table — (DOCX) [file pone.0340854.s002.docx]

| **Anthropology Plus** |
| --- |
| ( spirituality or spiritual needs or spiritual care or spiritu* ) AND ( Canada or Canadian or Canadians or in Canada ) AND ( health or wellbeing or well being or well-being or quality of life or wellness or physical health or social health or spiritual health or intellectual health or emotional health or financial health or environmental health or occupational health or intellectual health )  Limiters - Document Type: article; Language: english; Peer Reviewed  Expanders - Apply equivalent subjects  Search modes - Boolean/Phrase |
| **APA PsyINFO** |
| (spirituality or spiritual needs or spiritual care or spiritu* ) AND (Canada or Canadian or Canadians or in Canada) AND (health or wellbeing or well being or well-being or quality of life or wellness or physical health or social health or spiritual health or intellectual health or emotional health or financial health or environmental health or occupational health or intellectual health)Limits applied Databases: APA PsycInfo® Limited by:Peer reviewed Record type:Journal, Journal Article Language:English |
| **PubMed** |
| ((spirituality or spiritual needs or spiritual care or spiritu*) AND (Canada or Canadian or Canadians or in Canada)) AND (health or wellbeing or well being or well-being or quality of life or wellness or physical health or social health or spiritual health or intellectual health or emotional health or financial health or environmental health or occupational health or intellectual health))Limiters: Full-text, Free full-text; English language |
| **ProQuest Databases** |
| (noft(spirituality) or noft(spiritual needs) or noft(spiritual care) or noft(spiritu*) ) AND (noft(Canadian) or noft(Canadians) or noft(in Canada)) AND (noft(health) or noft(wellbeing) or noft(well being) or noft(well-being) or noft(quality of life) or noft(wellness) or noft(physical health) or noft(social health) or noft(spiritual health) or noft(intellectual health) or noft(emotional health) or noft(financial health) or noft(environmental health) or noft(occupational health) or noft(intellectual health)) Limiters: Peer-reviewed; English language |
| **Web of Science** |
| TS=((((spirituality or spiritual needs or spiritual care or spiritu*) AND (Canada or Canadian or Canadians or in Canada)) AND (health or wellbeing or well being or well-being or quality of life or wellness or physical health or social health or spiritual health or intellectual health or emotional health or financial health or environmental health or occupational health or intellectual health)): Limiters: English language; Journal Article |
| **EBSCO Host Databases** |
| ((spirituality or spiritual needs or spiritual care or spiritu*) AND (Canada or Canadian or Canadians or in Canada)) AND (health or wellbeing or well being or well-being or quality of life or wellness or physical health or social health or spiritual health or intellectual health or emotional health or financial health or environmental health or occupational health or intellectual health))  Limiters - Peer Reviewed; Publication Type: Academic Journal; Publication Type: Academic Journal; Language: English  Expanders - Apply equivalent subjects  Search modes - Boolean/Phrase |
| **Scopus** |
| ( ( spirituality OR spiritual AND needs OR spiritual AND care OR spiritu* ) AND ( canada OR canadian OR canadians OR in AND canada ) AND ( health OR wellbeing OR well AND being OR well-being OR quality AND of AND life OR wellness OR physical AND health OR social AND health OR spiritual AND health OR intellectual AND health OR emotional AND health OR financial AND health OR environmental AND health OR occupational AND health OR intellectual AND health ) ) AND ( LIMIT-TO ( DOCTYPE , "ar" ) ) AND ( LIMIT-TO ( LANGUAGE , "English" ) ) AND ( LIMIT-TO ( SEARCH WITHIN , "Article title, Abstract, Keywords" ) ) |
| **EMBASE** |
| ( (spirituality or spiritual) and needs) or spiritual) and care) or spiritu*) and (canada or canadian or canadians or in) and canada) and (health or wellbeing or well) and being) or well-being or quality) and of and life) or wellness or physical) and health) or social) and health) or spiritual) and health) or intellectual) and health) or emotional) and health) or financial) and health) or environmental) and health) or occupational) and health) or intellectual) and health)).mp. [mp=title, abstract, heading word, drug trade name, original title, device manufacturer, drug manufacturer, device trade name, keyword heading word, floating subheading word, candidate term word] Limit to English Language |
| **MEDLINE (through OVID)** |
| ((spirituality or spiritual needs or spiritual care or spiritu*) and (Canada or Canadian or Canadians or in Canada) and (health or wellbeing or well being or well-being or quality of life or wellness or physical health or social health or spiritual health or intellectual health or emotional health or financial health or environmental health or occupational health or intellectual health)).mp. [mp=title, book title, abstract, original title, name of substance word, subject heading word, floating sub-heading word, keyword heading word, organism supplementary concept word, protocol supplementary concept word, rare disease supplementary concept word, unique identifier, synonyms, population supplementary concept word, anatomy supplementary concept word] Limit to English Language |
| **CINAHL** |
| AB ( spirituality or spiritual needs or spiritual care or spiritu* ) AND AB ( Canada or Canadian or Canadians or in Canada ) AND AB ( health or wellbeing or well being or well-being or quality of life or wellness or physical health or social health or spiritual health or intellectual health or emotional health or financial health or environmental health or occupational health or intellectual health )  Search modes - Boolean/Phrase |

| **Hand Searching on Google Scholar** |
| --- |
| religion OR spirituality, wellbeing OR health, canada |
