# Supplementary material for: A scoping review and thematic analysis of the landscape of spiritual health and spirituality in Canada
Source: PLoS One. 2026 Feb 20;21(2):e0340854. doi: 10.1371/journal.pone.0340854 (PMC12923021; doi:10.1371/journal.pone.0340854)
Supplement: S3 Fig — (PDF) [file pone.0340854.s003.pdf]

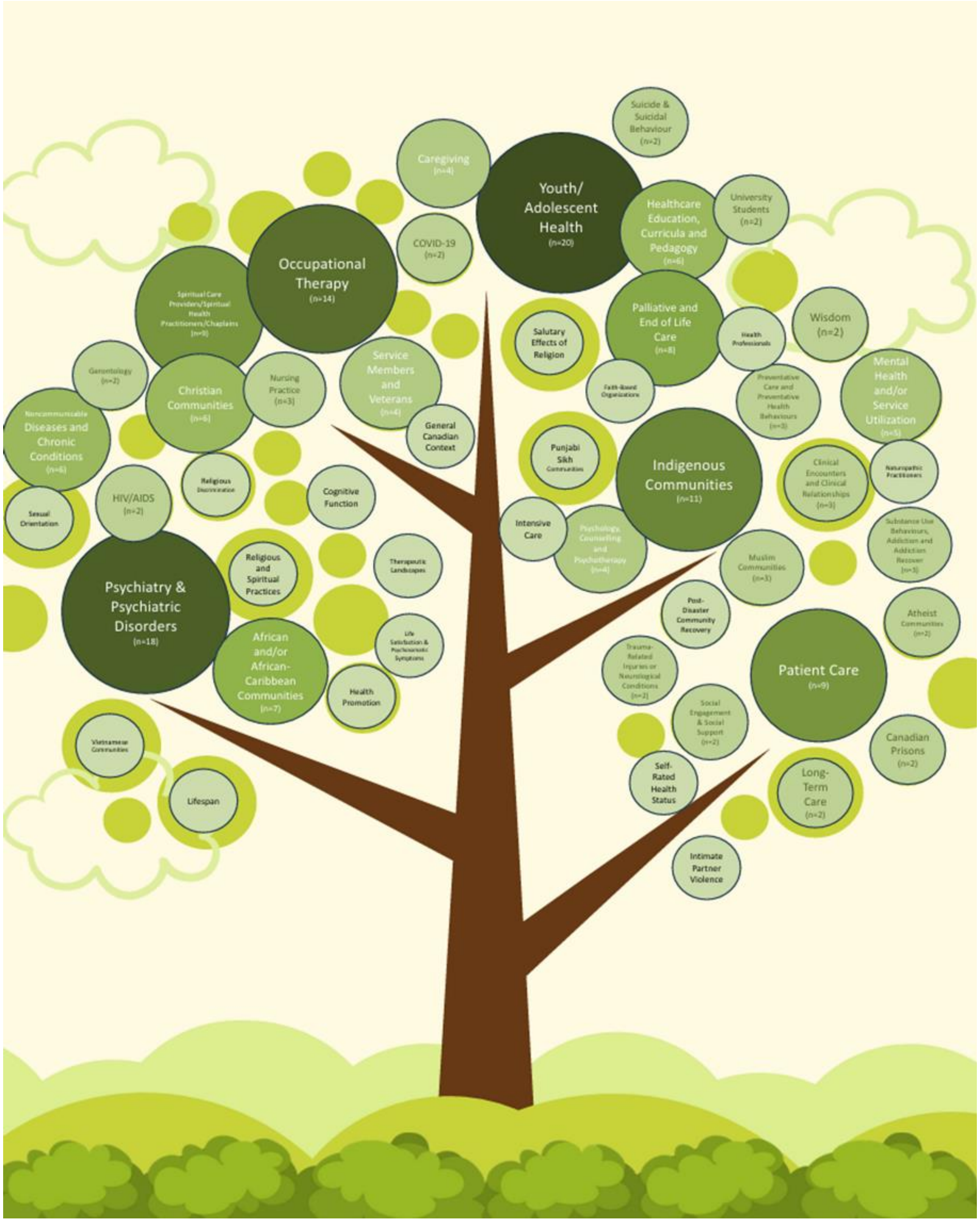
Suicide & Suicidal Behaviour (n=2)
Caregiving (n=4)
Youth/ Adolescent Health (n=20)
Healthcare Education, Curricula and Pedagogy (n=6)
University Students (n=2)
COVID-19 (n=2)
Occupational Therapy (n=14)
Spiritual Care Providers/Spiritual Health Practitioners/Chaplains (n=9)
Salutary Effects of Religion
Palliative and End of Life Care (n=8)
Health Professionals
Wisdom (n=2)
Gerontology (n=2)
Christian Communities (n=6)
Nursing Practice (n=3)
Service Members and Veterans (n=4)
Faith-Based Organizations
Preventative Care and Preventative Health Behaviours (n=3)
Mental Health and/or Service Utilization (n=5)
Noncommunicable Diseases and Chronic Conditions (n=6)
General Canadian Context
Punjabi Sikh Communities
Indigenous Communities (n=11)
Clinical Encounters and Clinical Relationships (n=3)
Naturopathic Practitioners
Religious Discrimination
HIV/AIDS (n=2)
Cognitive Function
Sexual Orientation
Intensive Care
Psychology, Counselling and Psychotherapy (n=4)
Substance Use Behaviours, Addiction and Addiction Recover (n=3)
Muslim Communities (n=3)
Religious and Spiritual Practices
Therapeutic Landscapes
Psychiatry & Psychiatric Disorders (n=18)
Post-Disaster Community Recovery
Atheist Communities (n=2)
Life Satisfaction & Psychosomatic Symptoms
African and/or African-Caribbean Communities (n=7)
Trauma-Related Injuries or Neurological Conditions (n=2)
Patient Care (n=9)
Health Promotion
Social Engagement & Social Support (n=2)
Canadian Prisons (n=2)
Vietnamese Communities
Self-Rated Health Status
Long-Term Care (n=2)
Lifespan
Intimate Partner Violence

Youth and Adolescent Health	20	Long-Term Care	2
Psychiatry and Psychiatric Disorders	18	Social Engagement and Social Support	2
Occupational Therapy	14	Suicide and Suicidal Behaviour	2
Indigenous Communities	11	Trauma-Related Injuries or Neurological Conditions	2
Patient Care	9	University Students	2
Spiritual Care Providers/Spiritual Health Practitioners/Chaplains	8	Wisdom	2
Palliative and End of Life Care	8	Cognitive Funnction	1
African and/or African-Caribbean Communities	7	Faith- Based Organizations	1
Christian Communities	6	General Canadian Context	1
Healthcare Education, Curricula, and Pedagogy	6	Health Promotionn	1
Noncommunicable Diseases and Chronic Conditions	6	Healthcare Professionals	1
Mental Health and/or Service Utilization	5	Intensive Care	1
Caregiving	4	Intimate Partner Violence	1
Psychology, Counselling, and Psychotherapy	4	Life Satisfaction and Psychosomatic Symptoms	1
Service Members and Veterans	4	Lifespan	1
Clinical Encounters and Clinical Relationships	3	Naturopathic Practitioners	1
Muslim Communities	3	Post-Disaster Community Recovery	1
Nursing Practice	3	Punjabi Sikh Communnities	1
Preventive Care and Preventive Health Behaviours	3	Religious and Spiritual Practices	1
Substance Use, Behaviours, Addition, and Addition Recovery	3	Religious Discrimination	1
Atheist Communities	2	Salutary Effects of Religion	1
Canadian Prisons	2	Self-Rated Health Status	1
COVID-19	2	Sexual Orientation	1
Gerontology	2	Therapeutic Landscapes	1
HIV/ AIDS	2	Vietnamese Communities	1
