# Supplementary material for: A scoping review and thematic analysis of the landscape of spiritual health and spirituality in Canada
Source: PLoS One. 2026 Feb 20;21(2):e0340854. doi: 10.1371/journal.pone.0340854 (PMC12923021; doi:10.1371/journal.pone.0340854)
Supplement: S2 File — (DOCX) [file pone.0340854.s005.docx]

1. Benson, P. L., Roehlkepartain, E. C., & Rude, S. P. (2003). Spiritual development in childhood and adolescence: Toward a field of inquiry*. Applied Developmental Science*, 7(3), 205–213. https://doi.org/10.1207/S1532480XADS0703_12
2. Boutros, H. M., Mina, M., Van Doorn-Harder, N., et al. (2024). The landscape of spiritual health and spirituality in Canada: A scoping review protocol. *PLOS ONE*, *19*(8), e0309294. <https://doi.org/10.1371/journal.pone.0309294>
3. Braun, V., & Clarke, V. (2019). Reflecting on reflexive thematic analysis. *Qualitative Research in Sport, Exercise and Health,* 11(4), 589–597. <https://doi.org/10.1080/2159676X.2019.1628806>
4. *CASC/ACSS*. (n.d.). CASC/ACSS. Retrieved December 15, 2024, from <https://www.spiritualcare.ca/>
5. Como, J. M. (2007). Spiritual practice: A literature review related to spiritual health and health outcomes. *Holistic Nursing Practice*, *21*(5), 224–236. <https://doi.org/10.1097/01.HNP.0000287986.17344.02>
6. De Diego-Cordero, R., Suárez-Reina, P., Badanta, B., et al. (2022). The efficacy of religious and spiritual interventions in nursing care to promote mental, physical and spiritual health: A systematic review and meta-analysis. *Applied Nursing Research*, *67*, 151618. <https://doi.org/10.1016/j.apnr.2022.151618>
7. Deneen, P. J. (2018). *Why liberalism failed* (Paperback edition). Yale University Press.
8. Furseth, I. 2018. *Religious Complexity in the Public Sphere: Comparing Nordic Countries*. New York: Palgrave Macmillan.
9. Herbert, D. E. J. (2011). Theorizing religion and media in contemporary societies: An account of religious ‘publicization.’ *European Journal of Cultural Studies*, *14*(6), 626–648. <https://doi.org/10.1177/1367549411419981>
10. King, M. B., & Koenig, H. G. (2009). Conceptualising spirituality for medical research and health service provision. *BMC Health Services Research, 9*(1), 1–7. https://doi.org/10.1186/1472-6963-9-116
11. Lasair, S. (2025). *Spirituality and Holistic Spiritual Health: Expanding Chaplaincy’s Theoretical Frame* (1st ed). Taylor & Francis Group.
12. Lee, S. J. C. (2002). In a secular spirit: strategies of Clinical Pastoral Education. *Health Care Analysis*, *10*(4), 339–356. <https://doi.org/10.1023/A:1023423125939>
13. Milbank, J. (1997). *The word made strange: theology, language, culture*. Blackwell Publishers.
14. Muhl, C., Cornish, E., Zhou, X. A., Mulligan, K., Bayoumi, I., Ashcroft, R., Ross-White, A., & Godfrey, C. (2025). Social Prescribing for Children and Youth: A Scoping Review. *Health & Social Care in the Community*, *2025*(1), 5265529. <https://doi.org/10.1155/hsc/5265529>
15. Muhl, C., Mulligan, K., Bayoumi, I., Ashcroft, R., & Godfrey, C. (2023). Establishing internationally accepted conceptual and operational definitions of social prescribing through expert consensus: a Delphi study. *BMJ Open*, *13*(7), e070184. <https://doi.org/10.1136/bmjopen-2022-070184>
16. Page, M. J., & Moher, D. (2017). Evaluations of the uptake and impact of the Preferred Reporting Items for Systematic reviews and Meta-Analyses (PRISMA) Statement and extensions: a scoping review. *Systematic Reviews*, *6*(1), 263. <https://doi.org/10.1186/s13643-017-0663-8>
17. Parker, C. (2018). Popular Religions and Multiple Modernities: A Non-Western Perspective. In W. I. April (Ed.), *Culture and Identity*. InTech. <https://doi.org/10.5772/intechopen.74411>
18. Pickett, W., King, N., Elgar, F. J., & Michaelson, V. E. (2025). Relative socio-economic position and meaning and purpose in life in adolescents: An intangible cost of social inequalities. *SSM-Population Health, 30, 101776.*
19. Prosman, H.-J. (2020). Radical Romanticism: postmodern polytheism in Richard Rorty and John Milbank. *International Journal of Philosophy and Theology*, *81*(1), 18–35. <https://doi.org/10.1080/21692327.2018.1542610>
20. Puchalski, C. M. (2014). The FICA Spiritual History Tool #274. Journal of Palliative Medicine, 17(1), 105–106. https://doi.org/10.1089/jpm.2013.9458
21. Puchalski, C., Ferrell, B., Virani, R., Otis-Green, S., Baird, P., Bull, J., Chochinov, H., Handzo, G., Nelson-Becker, H., Prince-Paul, M., Pugliese, K., & Sulmasy, D. (2009). Improving the Quality of Spiritual Care as a Dimension of Palliative Care: The Report of the Consensus Conference. *Journal of Palliative Medicine,* 12(10), 885–904. <https://doi.org/10.1089/jpm.2009.0142>
22. Roehlkepartain, E. C., Benson, P. L., Scales, P. C., Kimball, L., & King, P. E. (2008). *With their own voices: A global exploration of how today’s young people experience and think about spiritual development.* Center for Spiritual Development in Childhood and Adolescence.
23. Taylor, C. (2024). *Cosmic connections: poetry in the age of disenchantment*. The Belknap Press of Harvard University Press.
24. Taylor, C. (2018). *The ethics of authenticity* (Second printing.). Harvard University Press.
25. Tricco, A. C., Lillie, E., Zarin, W., et al. (2018). PRISMA Extension for Scoping Reviews (PRISMA-ScR): Checklist and Explanation. *Annals of Internal Medicine*, *169*(7), 467–473. <https://doi.org/10.7326/M18-0850>
26. Walton, M. N. (2012). Assessing the Construction of Spirituality: Conceptualizing Spirituality in Health Care Settings. *Journal of Pastoral Care & Counseling: Advancing Theory and Professional Practice through Scholarly and Reflective Publications*, *66*(3), 1–16. <https://doi.org/10.1177/154230501206600307>
27. Watson, J. (2003). Preparing spirituality for citizenship. *International Journal of Children’s Spirituality, 8*(1), 9–24. https://doi.org/10.1080/13644360304641
